# Discordant evolution of mitochondrial and nuclear yeast genomes at population level

**DOI:** 10.1186/s12915-020-00786-4

**Published:** 2020-05-11

**Authors:** Matteo De Chiara, Anne Friedrich, Benjamin Barré, Michael Breitenbach, Joseph Schacherer, Gianni Liti

**Affiliations:** 1 Université Côte d’Azur, CNRS, INSERM, IRCAN, Nice, France; 2 Université de Strasbourg, CNRS, GMGM UMR 7156, F-67000 Strasbourg, France; 3 Fachbereich Biowissenschaften, Universität Salzburg, Salzburg, Austria; 4 Institut Universitaire de France (IUF), Paris, France

**Keywords:** Yeast, Mitochondrial genome, Genome evolution, Molecular phylogeny

## Abstract

**Background:**

Mitochondria are essential organelles partially regulated by their own genomes. The mitochondrial genome maintenance and inheritance differ from the nuclear genome, potentially uncoupling their evolutionary trajectories. Here, we analysed mitochondrial sequences obtained from the 1011 *Saccharomyces cerevisiae* strain collection and identified pronounced differences with their nuclear genome counterparts.

**Results:**

In contrast with pre-whole genome duplication fungal species, *S. cerevisiae* mitochondrial genomes show higher genetic diversity compared to the nuclear genomes. Strikingly, mitochondrial genomes appear to be highly admixed, resulting in a complex interconnected phylogeny with a weak grouping of isolates, whereas interspecies introgressions are very rare. Complete genome assemblies revealed that structural rearrangements are nearly absent with rare inversions detected. We tracked intron variation in *COX1* and *COB* to infer gain and loss events throughout the species evolutionary history. Mitochondrial genome copy number is connected with the nuclear genome and linearly scale up with ploidy. We observed rare cases of naturally occurring mitochondrial DNA loss, *petite*, with a subset of them that do not suffer the expected growth defect in fermentable rich media.

**Conclusions:**

Overall, our results illustrate how differences in the biology of two genomes coexisting in the same cells can lead to discordant evolutionary histories.

## Background

Mitochondria are pivotal in eukaryotic cells, providing chemical energy in the form of ATP through oxidative phosphorylation [1], but also fulfilling a multitude of other critical functions including lipid and amino acids metabolism as well as heme and nucleotide synthesis [2]. These fundamental organelles share a common origin in eukaryotes deriving from ancestral α-proteobacterial symbiont [3] and are present in the vast majority of eukaryotes [4, 5]. Since then, mitochondrial genomes have undergone a drastic size reduction by gene transfer to the nucleus [6, 7], although they evolved in a large variety of sizes and gene content across species [8, 9]. Despite having their own genomes and translation machinery, mitochondria nevertheless remain strongly interconnected with the host cell. Several proteins are encoded at nuclear level, but they are relocalized into the mitochondria once matured. For example, eukaryotic ATP synthase subunits are encoded by both nuclear and mitochondrial genomes. Consequently, the products of nuclear encoded subunits need to be imported into the organelles and assembled in a coordinate manner into functioning structures [10]. The tight interplay between genes coded by nuclear and mitochondrial genomes has thus driven the coevolution of these interacting proteins, and incompatibilities between mitochondrial and nuclear alleles may contribute to reproductive barriers between species [11] or populations [12].

The mitochondrial DNA (mtDNA) has been widely used to infer phylogenetic relationships [13–16]. However, mtDNA phylogenies can vary from those generated from nuclear genome [17]. Mitochondrial genome is usually found in high copy number with multiple copies in each organelle [18]. Mutation rate of mtDNA often differs from the one of the nuclear DNA, and it can be significantly lower (plants and fungi) or higher (animals) [19]. In addition, mtDNA does not follow a Mendelian inheritance, since its replication and partition are not directly linked to the cell cycle and transmission is typically uniparental (maternal, in the vast majority of higher eukaryotes), eliminating the potential for sexual recombination. Cases of paternal or biparental inheritance have nevertheless been reported in some species [20, 21], although mtDNA heterogeneity does not persist. For instance, as in *Mytilidae* mussel species, where paternal mitochondria are only transmitted to the gonads of male offspring [22] and in ascomycetes yeasts where mitochondrial heteroplasmy, while being generated at the mating, is rapidly lost within the first few mitotic cycles [23].

Deleterious mutations that impair mitochondrial functions often cause severe and untreatable diseases [24]. Given the difficulty to engineer mtDNA in human cell lines, model genetic systems have paved the way for functional characterization. In particular, the budding yeast *Saccharomyces cerevisiae* has been widely used to study the phenotypic effect of mtDNA mutations [25–27]. *S. cerevisiae* mtDNA is unusually large, 85 kb; made of mostly non-coding A+T-rich sequence; and organized as either circular monomer or head-to-tail tandem-repeated linear structures [23]. The *S. cerevisiae* mtDNA contains eight genes encoding for three subunits of the ATP synthase complex (*ATP6*, *ATP8*, *ATP9/OLI1*), the apocytochrome b (*COB*), three subunits of the cytochrome c oxidase complex (*COX1*–*3*) and a ribosomal protein (*VAR1/RPS3*). *COB* and *COX1* contain a variable presence/absence pattern of introns, also present in other *Saccharomyces* species [28]. *COX1* introns are highly variable, reflecting mobility, while *COB* introns are mostly fixed, with edges of mobile introns that represent mutational hotspots [29]. In addition to the eight canonical protein-coding genes, the *S. cerevisiae* mitochondrial genome contains additional functional elements that include two ribosomal RNAs (*rnl* and *rns*), 24 tRNA and several replication-like origin [30]. The *rnl* 21S mitochondrial ribosomal subunit carries an intron, named *OMEGA*, which encode for a homing endonuclease (*F-SceIV*). Additional non-canonical ORFs are *RF1*, *RF2* and *RF3*. *RF1* and *RF3* encode for other two homing endonucleases (*F-SceIII* and *F-SceI*, respectively) and, together with *F-SceIV*, influence the mitochondrial genome stability by increasing the recombination rate [31, 32]. Additional invasive elements are the GC clusters within canonical and non-canonical genes. In particular, *F-SceI*, *RF2*, *F-SceIII* and *VAR1* are known to contain GC clusters. These clusters, at least in *VAR1*, can act as byp-like elements causing jumps in protein translation, yet maintaining the protein production [33]. In contrast, GC clusters in the endonuclease genes often cause frame shifts that inactivate the protein. GC clusters have also been shown to promote recombination within the GC-rich palindromes [34]. Recombination can occur between yeast mitochondria during the transient heteroplasmic phase following the mating. However, the two parental organelles are kept physically separated and can only be in contact and recombine on a limited interaction surface [35]. Nevertheless, recombination is considered to be a critical mechanism for mitochondrial inheritance [36] with profound effect on patterns of mitochondrial genetic variation [21, 37, 38].

The S288C laboratory genetic background contains approximately 20 copies of mitochondrial genomes, and 5% of non-essential genes are required for mtDNA maintenance [39]. Cells with mutated or without mitochondrial genomes are referred to as ‘petite’ given their slow growth phenotype in complete media and are unable to grow on non-fermentable carbon sources. Although *S. cerevisiae* is a leading model for molecular and genomics studies [40–42], most studies have focused on a small number of laboratory derivative isolates, whose genetic and phenotypic features have been shaped by laboratory manipulations [43]. Indeed, high mitochondrial genome instability, which promote petite formation, is a hallmark of *S. cerevisiae* laboratory strains [44].

The release of mitochondrial genomes of several isolates [45, 46] offered an opportunity for mitochondrial population surveys [28]. We recently released high coverage genome sequence for a panel of 1011 natural strains, isolated from both anthropic and wild niches [47]. The nuclear genome phylogeny revealed a strong population stratification with 26 separated lineages and three groups of outbred mosaic strains. These lineages were further partitioned in domesticated and wild lineages based on the ecological origin of the isolates. Nuclear genome diversity is different between these two classes of isolates, with wild lineages having a higher number of SNPs, while domesticated showing higher genome content variation. Here, we examined mitochondrial genome variation across the 1011 *S. cerevisiae* collection to trace the events that shaped their evolution and compare them with evolutionary patterns observed in the nuclear genomes. We detected strong admixture of the mtDNA, which cannot be fully captured by phylogenetic trees. The mitochondrial genomes have higher genetic variation compared to their nuclear genome counterparts with weak concordance between the two phylogenetic topologies. We observed high variation in mtDNA copy number and identified several natural petite isolates, with some that surprisingly do not show the expected growth rate defects in rich media.

## Results and discussion

### Mitochondrial genetic diversity across *S. cerevisiae* population

We explored 1011 *S. cerevisiae* sequenced isolates [47] to investigate the intraspecific mitochondrial genome diversity and evolution. Since mitochondrial genomes include variable long AT-rich intergenic regions that are difficult to compare, we first focused on the eight mitochondrial coding DNA sequences (CDSs). From 698 de novo genome assemblies, we collected the eight complete CDSs. Out of these, 553 isolates also had a complete or nearly complete mitochondrial sequence. A subset of 353 genome sequences did not have any ambiguous base across the CDSs (Additional file 1: Figure S1, Additional file 2: Table S1). We estimated the global genetic diversity by the average pairwise divergence *π*. Overall, we observed lower diversity in the coding nuclear (*π* ~ 0.003) [47] compared to the mitochondrial sequences (*π* ~ 0.0085, Additional file 2: Table S2), which contrasts to what was previously observed for other yeast species (Additional file 1: Figure S2). This opposing trend, more similar to the pattern observed in animal rather than in fungi [19, 48], is consistent with *S. cerevisiae* experienced rapid evolution of mitochondrial genes after the whole genome duplication [49].

We observed sharp genetic divergence differences of the nuclear and mitochondrial genomes among wild and domesticated isolates. In wild clades, despite higher nuclear divergence (up to 1.1% at CDS level), the mitochondrial CDS genetic distance reaches its maximum of ~ 0.6% at ~ 0.4% of nuclear divergence and plateau afterwards. In contrast, mitochondrial sequence divergence between domesticated clades have a larger increase, reaching its maximum at lower nuclear divergences (Additional file 1: Figure S3). This difference in variation is observed across all the mitochondrial CDSs whose values of *π* are systematically higher in domesticated compared to wild isolates.

**Fig. 1:**
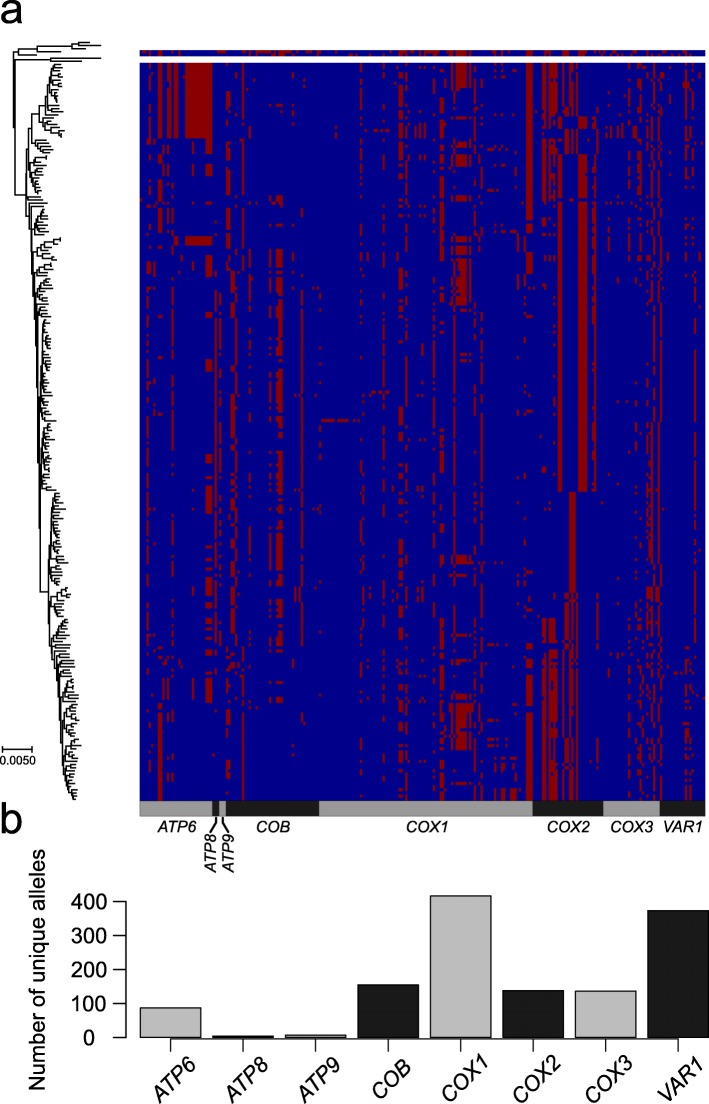
Allele distribution across mitochondrial CDSs. **a** Distribution of major (blue) and minor (red) alleles for the 259 polymorphic positions in the 234 *S. cerevisiae* unique complete profiles (which include 353 isolates). Profiles are ordered according to their phylogenetic relationship using the phylogenetic neighbour-joining tree (left side). **b** The numbers of unique alleles for each mitochondrial CDS show a dramatic difference between genes

The shortest CDSs, *ATP8* and *ATP9*, have the lowest proportion of polymorphic sites (~ 2%) and lowest values of *π* (0.003 or less) and lack non-synonymous mutations. In contrast, *COX1* and *COX2* are highly polymorphic. Although *COX1* has the highest polymorphic sites (8%), *COX2* has the highest *π* value (0.0163, Table 1). We used the discriminant analyses of principal component (DAPC) [50] to evaluate the contribution of specific genes to classify mitochondrial ‘haplotypes’ and population clustering. We quantified that *ATP6* and *COX2* respectively account for 38% and 28% of population clustering. This observation supports the widespread usage of *COX2* in mitochondrial phylogeny (Fig. 1a) [37, 38, 51, 52].

**Table 1:** Genetic diversity of mitochondrial CDSs

	Number of complete CDSs	Length of CDS in S288c	Length of alignment	Number of polymorphic sites	InDel positions	Pi	dN/dS (median value)
*ATP6*	933	780	780	41	0	0.0108	0.1503
*ATP8*	971	147	147	3	0	0.0034	0
*ATP9*	951	231	231	4	0	0.0013	0
*COB*	774	1158	1158	44	0	0.0048	0.0753
*COX1*	737	1605	1605	129	0	0.0088	0.1034
*COX2*	909	756	756	45	0	0.0163	0.0288
*COX3*	935	810	810	36	0	0.0072	0.0222
*VAR1*	453	1197	1302	82	190	0.0070	0.2500

Next, we generated a non-redundant allele database. We observed a variable number of distinct CDSs alleles (Fig. 1b), resulting in high proportion of unique allelic profiles (234 out of 353 isolates, Additional file 1: Figure S1). We used these non-redundant allelic profiles as a proxy to investigate the mitochondrial genome distribution in the population. Globally, we observed a poor overlap between the mitochondrial and nuclear genome phylogenetic lineages [47] with few exceptions that include near-to-clonal nuclear genome lineages, having specific mitochondrial profiles. These exceptions include a Sake subclade, the two clinical Wine/European subclades (Y′ amplification and *S. boulardii*), the North American and the reproductively isolated Malaysian clades [53]. In contrast, the mixed origin clade [47], which has highly diverse ecological (e.g. bakeries, beer, plants, animal, water, clinical sample) and geographical origins (e.g. Europe, Asia, Middle East, America), shows low mitochondrial intra-clade difference despite substantial nuclear genome variation (Additional file 1: Figure S4). Indeed, across the mixed origin clade, only very similar profiles of mitochondrial genes segregate, with variants limited to *COX1* and *VAR1*, resulting in very low *π* (0.00008) compared to other clades (~ 0.001, Additional file 2: Table S2).

The *VAR1* gene is a particularly variable, highly AT rich and prone to non-synonymous mutations and indels. These indels mostly represent GC-rich byp-like elements able to cause jumps in protein translation in other yeast species [33]. Two positions were described, one named ‘common’ and another downstream with GC cluster in inverted orientation [54]. We identified 35 allelic variants of *VAR1* gene harbouring these two clusters in 117 isolates, mainly belonging to the mosaic groups (*N* = 52) (Additional file 2: Table S1). While most of the reported cases harboured the GC cluster either in the common (*N* = 91, accounting for 18 different *VAR1* alleles), or in both positions (*N* = 6, across 4 *VAR1* alleles) [54], a large fraction of the observed allelic variants here only harboured the GC cluster in the second site (*N* = 19, across 13 *VAR1* alleles). We also discovered two novel variants, one with GC cluster at the common position but in inverted orientation (2 isolates) and the second with the GC cluster in tandem duplication at the second position (3 isolates).

In addition to the canonical ORFs, we characterized the four non-canonical ORFs *F-SceIV* (*OMEGA* intron), *F-SceI* (*RF3*), *RF2* and *F-SceIII* (*RF1*) [31, 32]. *F-SceIV* is relatively uncommon in the population (198 isolates), while *F-SceI*, *RF2* and *F-SceIII* are more spread (447, 542 and 477 isolates, respectively). These three ORFs are known to contain GC clusters, which often introduce frame shifts in the sequences. In *F-SceIII*, we identified three GC clusters positions. The first position is particularly rare (43 isolates), and in two cases, the GC cluster is truncated. The other two GC clusters are much more abundant (in 277 and 206 isolates, respectively). We identified 6 distinct GC cluster positions in both *RF2* and *F-SceI* (see Additional file 2: Table S1). Altogether, these results uncovered high variability of mitochondrial sequence across the *S. cerevisiae* natural population.

### Extensive admixture of mitochondrial genomes

**Fig. 2:**
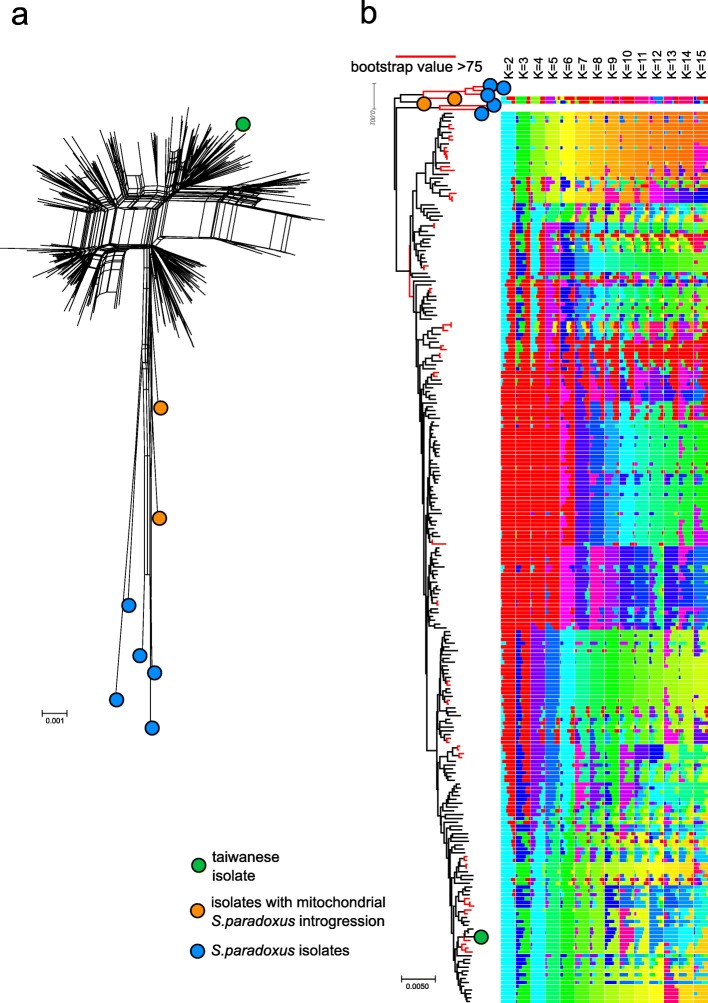
Complex mitochondrial genome phylogeny. Only *S. cerevisiae* isolates with complete CDS data have been used (*N* = 353) in addition to five *S. paradoxus* isolates used as an outgroup. **a** Phylogenetic network of non-redundant concatenated CDS sequences (*N* = 237 profiles) produced a highly intertwined network driven by recombination with few groups of closely related strains. **b** The rooted tree (left) shows a weak topology with few nodes (red) with bootstrap values over 75. ADMIXTURE analysis of genomic components (right) with *K* ranging from 2 to 15 confirms the high degree of mosaicism. The highly divergent Taiwanese lineage (green dot) is not divergent to the other lineages in contrast to the nuclear genome phylogeny

We investigated the mitochondrial genome population structure using the eight concatenated CDS to calculate the phylogenetic network using SPLITSTREE [55]. The dataset comprises 239 non-redundant CDS profiles, with 234 *S. cerevisiae* isolates with complete CDS sequences and five *S. paradoxus* representatives [56] as outgroups. The resulting intertwined network shows a strong interconnectivity of the sequences, underlying frequent historical recombination (Fig. 2a). In contrast, classical phylogenetic trees are unable to consistently group the isolates (Fig. 2b). Using ADMIXTURE [57], we observed that the opposite edges of the trees fall in the same population for low *K* values (*K* = 2–3), further underlying a poor grouping.

Mitochondrial population structure appears to poorly reflect the clustering obtained from the nuclear genome. For example, the early divergent Taiwanese lineage based on nuclear genome does not show higher sequence distance. However, isolates belonging to the mosaic groups of the *S. cerevisiae* population show the highest degrees of admixture, indicating that outbreeding has impacted both mitochondrial and nuclear genomes.

We then calculated the coefficient of concordance ‘W’ using the congruence among distance matrix (CADM) metric [58] to be 0.79, with 0 indicating complete disagreement and 1 complete agreement between distance matrixes. This value indicates a relatively good concordance between the phylogenic networks of mitochondrial and nuclear genomes. This is likely driven by isolates with very close mitochondrial sequence often also having similar nuclear genome sequence, while the main branches of the mitochondrial tree are discordant. We further compared phylogenetic trees and networks based on concatenated sequences derived from the 8 mitochondrial CDSs and 8 nuclear genes previously used for phylogenetic studies [59, 60] in a selection of 14 isolates (Additional file 1: Figure S5). Consistently, mitochondrial sequences resulted in a wider network, implying a less defined phylogenetic structure and more pronounced admixture, with early branching lineages falling within the worldwide non-Chinese lineages. Overall, our results highlight a pronounced separation in evolutionary histories of the two coexisting genomes, and the extensive mitochondrial genome admixture provides additional support to its mitochondrial inheritance requiring recombination-driven replication [34, 61, 62].

### Interspecies introgressions of mtDNA are rare

**Fig. 3:**
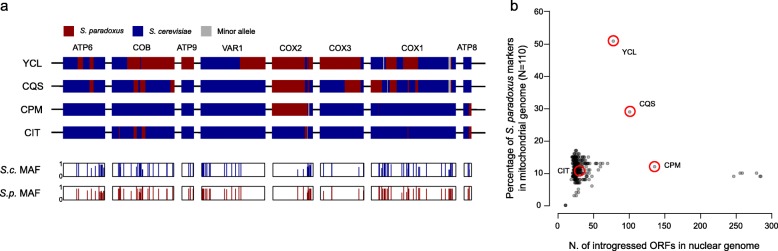
Rare *S. paradoxus* introgressions. **a** Polymorphic markers between *S. cerevisiae* and *S. paradoxus* across the mitochondrial CDSs were used to identify introgression events. Introgression boundaries are set as the midpoint between markers. The two bottom rows indicate the frequency in the population of the major or consensus allele (AF), in the specific position and species. **b** Number of introgressed ORFs in the nuclear genome does not correlate with the percentage of genetic markers of *S. paradoxus* in mitochondrial CDS. Only isolates with complete unambiguous CDS data were included (*N* = 353). Position of isolate reported in **a** is circled in red

We recently described four clades (namely Alpechin, Mexican Agave, French Guiana and Brazilian bioethanol) with abundant *S. paradoxus* interspecies introgressions in the nuclear genome [47]. We analysed the mitochondrial CDSs to search for introgressed alleles. The four clades with abundant nuclear genome introgressions did not show any *S. paradoxus* mitochondrial alleles. Nevertheless, two isolates from America (CQS, YCL) and one from Africa (ADE), all genetically related to the French Guiana and the Mexican Agave clades, harbour two distinct patterns of *S. paradoxus* mitochondrial introgressions. We retrieved the complete CDS set for two of them (CQS and YCL), while the third (ADE) is incomplete but very close to YCL. The mitochondria introgression in YCL (YJM1399) strain was already reported, but no further analyses were presented [28]. We generated a set of polymorphic markers (methods), to accurately identify the introgression boundaries. The *S. cerevisiae* major alleles were identified from the 1011 isolates, whereas for *S. paradoxus*, they were derived from 23 North American isolates for which full chromosome sequence were available [21, 56]. Eurasian *S. paradoxus* isolates were not included because of their similarity with *S. cerevisiae* sequences, likely due to an ancient introgression event from *S. cerevisiae* to *S. paradoxus* [21, 56, 63]. We generated a catalogue of 110 polymorphic positions and derived different alleles between the two species. Several genes in these two isolates were catalogued either as partially or fully introgressed (Fig. 3a). Since the frequency of some alleles is close to 50% and often the less common allele of one species is the more common allele of the second one, there is a chance of calling false-positive introgressions. Nevertheless, long consecutive series of *S. paradoxus* marker in the *COB*, *ATP9*, *COX1*, *COX2* and *COX3* genes in YCL, as well as those in the *COB*, *COX1*, *COX2* and *COX3* genes in CQS, are likely to be genuine. The absence of traces of introgression in *S. cerevisiae* isolates from Europe could be explained by the higher sequence similarity with European *S. paradoxus*, which prevent the detection. However, introgressions between *S. cerevisiae* and European *S. paradoxus* isolates could also be prevented by the non-collinearity in the structure of their mitochondrial genomes that likely impair the recombination [56].

We further extend the analysis of the 110 polymorphic sites to 353 isolates with fully assembled CDS. We observed additional potential cases of mitochondrial introgressions. The isolate YCL mitochondrial sequence harbours over 50% of *S. paradoxus* markers, possibly indicating a recombinant genome derived from a recent transfer event. In addition, a small number of *S. paradoxus* markers are found in each *S. cerevisiae* isolate, perhaps due to incomplete lineage sorting. Overall, the number of *S. paradoxus* markers in the mitochondrial genomes does not correlate with the number of introgressed ORFs in the nuclear genomes (Fig. 3b), suggesting that the interspecies gene flows were independent due to distinct origin and/or fate.

### Introns gain and loss during evolution and dispersal

**Fig. 4:**
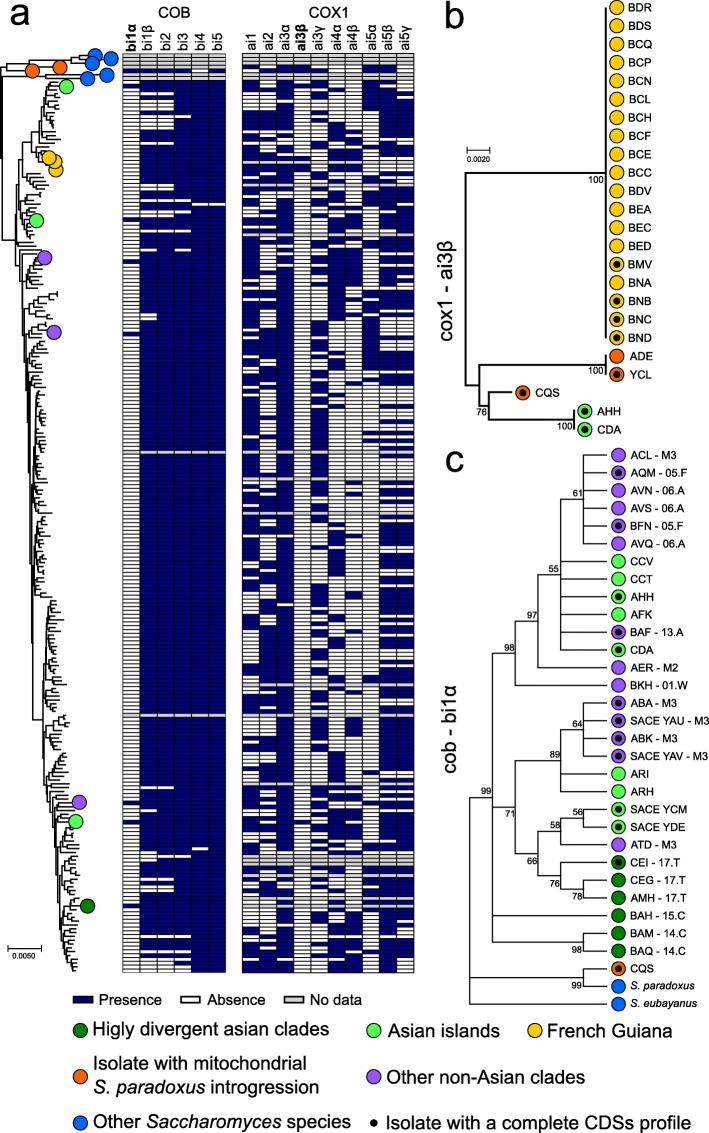
Intron phylogeny underlies both loss and gain events. **a** The distribution of intron presence and absence is not consistent with the mitochondrial tree phylogeny. The rare introns bi1α and ai3β are highlighted (bold); intron ai4γ was not found in the sequenced collection and not shown. **b** Only 4 non-redundant sequences have been found for cox1 ai3β intron. Their sequences are unrelated to other *Saccharomyces* species, which could not be used for rooting. The peculiarity of the distribution of this intron could suggest a lineage-specific gain event. **c** Rooted tree of the *COB* intron bi1α using *S. paradoxus* and *S. eubayanus* sequences as an outgroup. Nodes with bootstrap values below 0.5 have been collapsed. Its presence in multiple highly divergent Asian lineages and in other *Saccharomyces* species is consistent with intron loss following the out-of-China dispersal. The isolate CQS, which harbours introgression in both nuclear and mitochondrial genome, also derive from *S. paradoxus* origin. This is compatible with the downstream exonic sequence, which also is introgressed

Two mitochondrial protein coding genes, *COB* and *COX1*, harbour introns at multiple sites, and we explored their presence-absence patterns in the whole 1011 isolate collection. *COX1* introns are found at varying frequencies (median 0.48) with highly variable presence-absence profiles (Fig. 4a). Intron patterns further support low variability within North American, Malaysian and mixed origin lineages (Additional file 1: Figure S6). In contrast, the groups of loosely related mosaics (M1, M2 and M3 clusters) show the lowest level of intron conservation, consistent with their admixed genetic backgrounds.

The *COX1* intron frequencies in the population are consistent with previous report [28], ranging from 26 to 86%. We identified a total of 103 different *COX1* intron combinations with two introns, ai4β and ai5α, that are never found together (ai4β is in 89 while ai5α in 85, out of 408 alleles). Given the linkage between these closely spaced intronic positions, they either raised in two ancestral populations or are unlikely to be brought together by recombination or the double presence is functionally incompatible. Two additional *COX1* introns, ai3β and ai4γ, are very rare in *S. cerevisiae* population. While aI4γ is also absent in most related *Saccharomyces* species, ai3β intron is present in all of them. The only occurrence of ai3β previously reported in *S. cerevisiae* was in the YCL isolate, which also contains *S. paradoxus* introgression around the intron position in *COX1*. However, although the ai3β intron is present in *S. paradoxus*, the ai3β intron sequence of YCL is closer to the one found in *Lachancea meyersii* [28]. In addition to the YCL allele, we found other three variants of ai3β, all related to the *Lachancea* sequence. Two variants are present in YCL and ADE isolates with abundant *S. paradoxus* introgressions, while the CQS strain has related version. Additional ai3β intron is present in two Asian isolates and in 19 French Guiana isolates, whose clade is highly introgressed from *S. paradoxus* (Fig. 4b). The presence of the ai3β intron among these highly introgressed lineages suggests separate lateral transfer events from *Lachancea*, although it cannot be ruled out that these introns where initially transferred from *Lachancea*, or a related genus, to *S. paradoxus* before the introgression occurred.

In contrast, the six *COB* introns are more uniformly present (frequencies ranging from 88 to 99%, Fig. 4a) with the only exception of the recently described bi1α [28] occurring at low frequency (~ 5%). Surprisingly, bi1α is common among the early-branching Asian clades [47]. Other isolates harbour it, mainly mosaic isolates, but segregate at low frequency in non-Asian clades. Its presence in several *Saccharomyces* outgroup species and in the *S. cerevisiae* early divergent lineages suggests a loss preceding or during the out-of-Asia dispersal. The intron could have been introduced again, from secondary contacts with bi1α-positive Asian lines. To test these hypotheses, we constructed a phylogenetic tree using all the bI1α intron sequences and outgroups (Fig. 4c). The bi1α phylogenetic tree shows more variants of Asian sequences compared to non-Asian ones, which mainly cluster in two groups stemming from separated branches of Asian introns, consistent with multiple separate regain events in the worldwide population.

Self-splicing introns have been associated to increased mutation frequencies at the boundary intron/exon [29]. We scanned the exonic sequences in a window of 70 nucleotides both upstream and downstream each intron in *COX1* and *COB*. Consistently, the highly mobile *COX1* introns are associated with higher frequency of alternative alleles in a 20-nucleotide window adjacent to insertion boundaries (Additional file 1: Figure S7).

### Structural rearrangements are rare in mitochondrial genomes

Next, we investigate the size and the presence of structural variation across the mitochondrial genomes. Considering the 250 circularized assemblies, the mitochondrial genome sizes range from 73,450 to 95,658 bp (Additional file 2: Table S3). As the gene content is entirely conserved between these isolates, this high size plasticity is driven by variability of the intergenic region (ranging from 45,254 to 69,807 bp) and the intron content (ranging from 7748 to 20,024 bp in size) (Additional file 1: Figure S8). Both factors are highly correlated to the total mitochondrial genome length (*r*^2^ 0.769 and 0.756, respectively; correlation-associated *p* values < 2.0E−04) (Additional file 1: Figure S9). Mitochondrial genome size is variable among isolates of the same lineage.

**Fig. 5:**
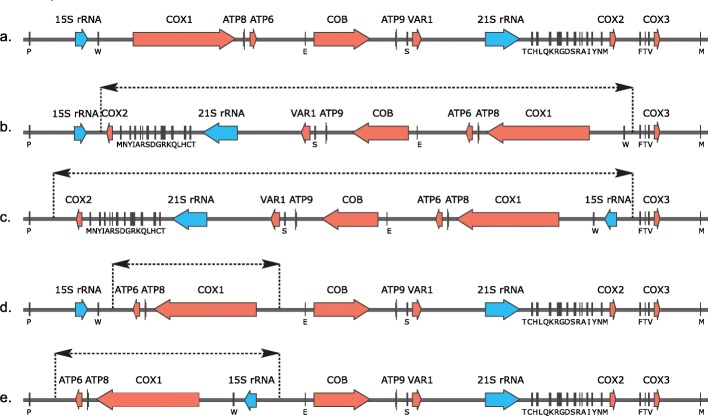
Structural variants in the mitochondrial genomes. Schematic of the mitochondrial genome organization annotated for protein-coding genes and rRNA and tRNA genes. The approximate breakpoint locations of the inversions are indicated by dotted lines. These mitochondrial genome organizations are related to different isolates. **a** S288C (shared by the vast majority of isolates). **b** AQI and BKI. **c** AIM, BNG and CFB. **d** CDN. **e** BDN

Synteny analysis across the 553 isolates with genome on single scaffold highlights four distinct genomic inversions (Fig. 5, Additional file 1: Figure S10). Two strains from the Wine/European and Ale beer lineages, BKI and AQT, share an inversion of the region that ranges from trnW to the *COX2* gene, while three closely related Wine/European strains (AIM, BNG and CFB) share a larger inversion that also encompasses the 15S rRNA gene (Fig. 5b, c). Inversions were also found in BDN (African beer) and CDN (Ecuadorean) and are related to regions ranging from the 15S rRNA or *COX1* genes, respectively, to the *ATP6* gene (Fig. 5d, e). All inversion boundaries map to highly repetitive AT-rich intergenic regions, which prevent their precise delimitation. Interestingly, all these inversions lead to the loss of a feature shared by most ascomycetous yeast, namely that all mitochondrial protein-coding genes are transcribed from the same DNA strand [64]. However, mitochondrial functions seem not to be impaired, as these isolates maintain their respiration capabilities.

Recent report suggested that the alteration of the gene order within yeast genera could be related to the mitochondrial genome size [65]. While the *Lachancea* and *Yarrowia* clades, with mitochondrial genome less than 50 kb, show high synteny across species [66, 67], the *Saccharomyces* clade (mitochondrial genome size > 65 kb) is more prone to rearrangements [65]. Indeed structural rearrangements were also detected in the mitochondrial genome of *S. paradoxus* [56]. Our results suggest that mtDNA structural variation can be tolerated, perhaps restricted to balanced events that do not alter the CDS copy number.

### Variation in mtDNA copy number reveal natural petite isolates

**Fig. 6:**
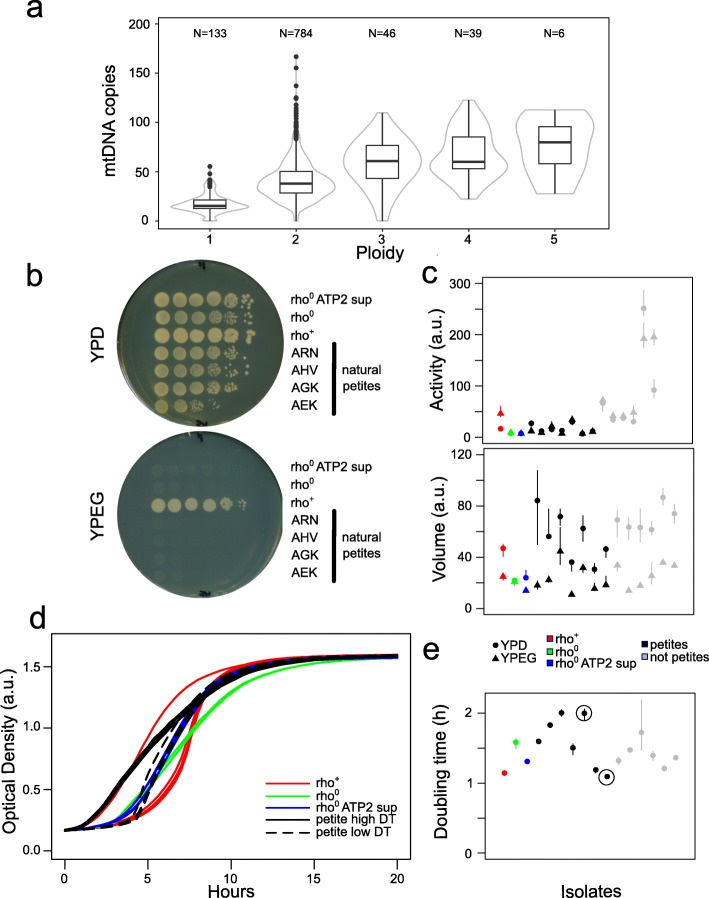
Natural variation in mitochondrial genome copy number. **a** Mitochondrial genome copy number linearly increase with the nuclear genome content. Fifteen natural petite isolates were detected. The number of isolates is indicated above the corresponding plot. **b** Spotting assay on non-fermentable carbon source (YPEG) confirms natural petite isolates (a subset of tested isolates is shown). **c** Mitochondrial activity (as membrane potential) is strongly altered by the lack of mitochondrial genome (black versus grey symbols), while the volume remains unaltered. **d** Growth curve variation of isogenic strains with normal mitochondria (rho^+^, red), petite (rho^0^, green) and petite harbouring the ATP2G1099T suppressing mutation (rho^0^ ATP2 sup, blue). Among natural petites, we can identify both isolates with high doubling time (DT, black solid line) and isolates with recovered growth rate, comparable to petite with suppressor mutations (black dashed line). **e** Generation times for isolates with different mitochondrial CN show at least two natural petite isolates that seem to have recovered normal growth rate on rich media. Growth curves for the circled isolates are shown in **d**

Mitochondrial copy number can dramatically affect phenotypes but is hard to measure with high-throughput methods. We estimated mtDNA copy number using the relative coverage of *ATP6*, *COX2* and *COX3*, which provide robust mapping. The number of mitochondrial genomes is generally constant across clades (Additional file 1: Figure S11), with no significant differences between domesticated and wild lineages, with a median of 18 mitochondrial genomes for each haploid nuclear genome. The variation is however particularly high across the population, reaching over 80 copies. As previously reported [68], the mtDNA copy number scales up with ploidy in a linear way, with diploid strains having around double number of mitochondrial genomes and triploid having three times the number, compared to haploid cells (Fig. 6a).

The presence of mitochondrial genomes is assumed to be the natural state of *S. cerevisiae* cells, which is defined as rho^+^. However, strains can lose mitochondrial functionality under different conditions either by accumulating mutations (rho^−^) or by complete loss (rho^0^) of the mitochondrial genome. These mutants are defined as ‘cytoplasmic petite’ (i.e. ‘small’) because they form small colonies in rich fermentable media due to their slow growth. Since the respiration-mediated ATP production is impaired in petite strains, they are unable to grow in non-fermentable carbon sources. We identified 15 potential natural petites isolates (Additional file 2: Tables S1 and S4) from coverage analysis and confirmed that they were unable to grow on non-fermentable carbon sources based media (Fig. 6b). All petite isolates appear to be rho^0^, with the exception of two rho^−^ isolates: ABM which retained *COB* gene and AHV which kept *ATP9* and *VAR1* genes (Additional file 2: Table S4). These rho^−^ isolates, with other five rho^0^ petite, were laboratory-derived haploid (*HO* deleted), and since strain manipulation could have caused their mitochondrial condition, they were excluded from further analyses. We examined a selection of four strains for mitochondrial activity (measured as membrane potential) and volume. We included as controls a wild-type rho^+^ strain and two derived rho^0^ variants with one wearing an additional mutation (*ATP2* G1099T), which partially restore growth in rich media (Michael Breitenbach, unpublished data). As expected, activity data show inability to grow on non-fermentable carbon sources (YPEG). There was, however, no significant variation between mitochondrial volume of wild-type and petites isolates, consistent with the essentiality of maintaining mitochondria also in petite strains (Fig. 6c, Additional file 2: Table S4). We investigated if these natural petites have doubling time defect by measuring growth curves in rich media (YPD). The petite strains showed different growth rates, with two of them having close to normal doubling time (Fig. 6d, e; Additional file 2: Table S4). These strains do not have neither *ATP2* G1099T nor *ATP3* G348T polymorphism that partially restore growth in rich media (Michael Breitenbach, unpublished data); hence, other compensatory mutations might have partially restored growth in these strains. We cannot rule out that the petite phenotype might have raised during laboratory manipulations; however, restoring near-to-normal growth in some of these isolates has likely required extensive propagation with large population sizes suggesting a more distant mtDNA loss event. Sporulation is known to be impaired in petite isolates [69], and consistently, all natural petite isolates do not sporulate.

## Conclusions

The *S. cerevisiae* yeast has long been considered as an organism with an almost clonal reproduction and strictly uniparental mitochondrial inheritance [34]. Nevertheless, the scenario emerging from our phylogenetic analyses revealed that outbreeding and recombination drive mitochondrial genome evolution. Quantitative variation between mitochondrial and nuclear genomes in admixture, population structures and sequence diversity underlies how differences in biology and selection of two genomes coexisting in the same cells can lead to highly discordant evolutionary trajectories. Furthermore, mitochondrial genomes are more refractory to interspecies introgressions, with only rare examples in the population. However, these rare events further support the contribution from both parental strains to the mitochondrial sequence, implying transient coexistence of unknown timespan. This situation can lead to incomplete lineage sorting, as suggested by the allele frequency distribution in *S. cerevisiae* and *S. paradoxus* mitochondrial genomes.

We observed a broad distribution of mitochondrial genome copy number across the population, of similar magnitude of variation generated in the knockout collection [39], which broadly scales up linearly with nuclear genome ploidy. The occurrence of natural petite strains, remarkably able to grow in fermentation media without detectable defects, demonstrates how variation in the nuclear genome can compensate the complete loss of mitochondrial genomes. Overall, these observations further reinforce the strong functional interplay between the two coexisting genomes despite their discordant evolutive trajectories. Future efforts will reveal the molecular details of such two-genome interactions and quantify their contribution toward the species phenotypic variation.

## Methods

### Mitochondrial genome assembly

We investigated a total of 1011 *S. cerevisiae* isolates from the 1002 Yeast Genome Project [47]. Among these, 84 isolates already had their complete mitochondrial sequence available [46]. For all other isolates, we identified mitochondrial scaffolds from whole genome assemblies through similarity searches with the BLAST suite of programmes [70], using the S288C mitochondrial sequence (EMBL: KP263414) as a query. In total, 905 mitochondrial assemblies could be retrieved, consisting of 1 to 27 scaffolds (Additional file 2: Table S5). Among them, 468 mitochondrial assemblies consist of a single scaffold longer than 70 kb and 166 could be circularized by Circlator [71] bringing the total number of circularized assemblies to 250. The final assemblies were linearized so that their start corresponds to the starting position of the S288C reference genome (Additional file 2: Table S3). As results, 698 assemblies had the full set of CDSs available, 353 of which without any ambiguous position and were hence used for the phylogenetic inferences.

### Genes and coding sequences

The sequences of the eight protein-coding genes (*ATP6*, *ATP8*, *ATP9*, *COB*, *COX1*, *COX2*, *COX3* and *VAR1*) were retrieved from the 1011 assemblies by performing BLAST similarity searches. The boundaries of the coding regions of *COX1* and *COB* genes were identified through pairwise global alignments between the gene sequences and the reference CDS, constructed with stretcher [72], and manually refined. The average pairwise diversity *π* was calculated with Variscan [73] while sequence divergence was calculated as the percentage of SNPs. Presences of omega intron and RF1, RF2 and RF3 endonuclease were identified by blast search using a cut-off of 90% of identity on 90% of the sequences. Query sequence were the following: omega intron SGD:S000007279, RF1 accession number KP263414.1 (positions 74495 to 75984), RF2 accession number X06706.1 (positions 52 to 1535) and RF3 accession number CP006487.1 (position 28758 to 30259).

### *S. paradoxus* marker identification

Polymorphic positions were extracted by aligning the isolate CDSs to the S288C ones running MUMmer v3.0 [74] both for the *S. cerevisiae* collection and a set of 23 North American *S. paradoxus* strains [21, 56]. The three American and Hawaiian *S. paradoxus* isolates were used while the European and Far East Asian isolates were removed due to their similarity to the *S. cerevisiae* mitochondrial sequence. SNPs were extracted with the command show-snps and the options –CrIT. For both species, each polymorphic position was evaluated to obtain the major allele for both species. All positions where the major alleles differed were retained to generate a marker list of 110 positions scattered across the genome. It is important to notice that, probably due to incomplete lineage sorting, in most of the cases, the less common allele of one of the two species corresponds to the more common allele of the other one, which introduces a certain amount of noise among the selected markers.

### Population structure

A non-redundant database of profiles of mitochondrial CDSs was built for the isolates with complete CDS sequences. Variant Call Format (VCF) file was created running snp-sites v2.3.3 [75] (snp-sites -v –o mitochondria.vcf ConcatenatedCDS.fasta) on the concatenation of the CDS genes multiple alignment. Plink v1.90 [76] (plink --vcf mitoCDS_conc.vcf --make-bed –out plink.out) was used to prepare the data to run ADMIXTURE v1.3.0 [57] software (admixture plink.out.bed K, with K from 2 to 15) .

The concatenations of CDS were also used to run SplitsTree v4.16.6 [55] and produce the phylogenetic network while the bootstrap of the NJ phylogenetic tree was produced by MEGA v7.0.26 [77].

### Identification of rearrangements

The gene coordinates were determined on the one-scaffold assemblies by running tRNAscan for tRNA and BlastN similarity searches for rRNA and protein coding genes. Structural rearrangements were detected by loss of synteny. The rearranged assemblies were further investigated through alignment with the reference mitochondrial genome with MUMmer v3.0 [74], nucmer was used to align the sequences and plots were generated with mummerplot.

### Copy number estimation

The number of copies of the mitochondrial genome was assessed by mapping, using BWA v0.7.15 [78] with the option –U 0. The reads of individual strains of three mitochondrial CDSs (*ATP6*, *COX2*, *COX3*) gave the most reliable copy number estimation. These CDSs were chosen for the lack of introns and their size. Samtools view was used to filter the results with the option –q 20. The copy number for haploid genome was estimated as the ratio between the average of the median coverage for the three mitochondrial CDS and the nuclear genome coverage (median of the median coverage for each chromosome). The estimated total copy number for each isolate was calculated as this copy number times the ploidy.

### Spotting assay

Cells were pre-grown overnight in liquid YPD before being diluted in water. For each 10-fold dilution, 5 μL was spotted either on YPD or YPEG (3% ethanol, 3% glycerol, 1% yeast extract, 2% peptones) agar plates. Plates were then incubated 48 h at 30 °C, and images were acquired with a Epson Perfection V330 scanner.

### Growth curves

A YPD overnight culture was 1:100 diluted into fresh YPD in a 96-well plate. Cells were incubated for 48 h at 30 °C in a plate reader (Tecan, Infinite F200 Pro). Optical density (600 nm) was monitored every 20 min. Four replicates were run for each strain. The PRECOG software [79] was used to calculate the doubling times.

### Mitochondrial activity and volume

Cells grown overnight in liquid YPD were diluted 40× in 200 μL of fresh YPD or fresh YPEG (1% yeast extract, 2% peptones, 3% ethanol, 3% glycerol) in a 96-well plate and incubated 5 h at 30 °C. Respectively 8 μL/30 μL of cells from YPD/YPEG were then transferred into 192 μL/170 μL of a HEPES 10 mM-glucose 2% solution. Samples were washed twice with HEPES-glucose and finally resuspended in 100 μL of mitochondrial staining solution (HEPES-glucose + 100 nM MitoTracker Green (Molecular Probes) + 20 nM MitoTracker Deep Red (Molecular Probes)) in a 96-well plate and incubated 30 min in the dark at 30 °C. MitoTracker Green is known to accumulate passively into the mitochondria proportionally to their volume, whereas MitoTracker Deep Red is known to accumulate into mitochondria proportionally to their membrane potential. The samples were analysed on a FACSCalibur using the HTS module. The relative mitochondrial volume was estimated by reading fluorescence in the FL-1 channel, whereas the relative mitochondrial activity was estimated by reading fluorescence in the FL-4 channel.

### Petite isolate sporulation activity

The 1011 sequenced collection was phenotyped for sporulation efficiency (De Chiara et al., in preparation). Isolates were pre-cultivated in yeast peptone dextrose (YPD; 2% dextrose, 1% yeast extract, 2% peptone, 2% agar) before being diluted 1:50 into 10 mL of pre-sporulation media (YPA; 2% potassium acetate, 1% yeast extract, 2% peptone) and grown 48 h at 30 °C (shaking = 250 rpm). Pre-sporulated cells were transferred to sporulation media (2% potassium acetate) into 250-mL flasks at a 5:1 volume/medium ratio and a final optical density (OD600) of 0.6. The flasks were kept at 23 °C and shaken at 250 rpm. To estimate sporulation efficiency, > 100 cells/sample were counted at 24- and 72-h post-transfer to sporulation medium using an optical microscope (Zeiss Axio Lab.A1). Sporulation efficiency was estimated as the number of asci divided by the total cell count.

## Supplementary information

**Additional file 1 **: **Figure S1** Dataset overview. Dataset overview for each clade (named as in Peter et al. 2018) for number of isolates with genome sequenced, complete CDSs assembled and non-redundant profiles. **Figure S2** Genetic diversity of nuclear and mitochondrial genes for three yeast species. Distribution of the π values of mitochondrial and nuclear protein coding genes for *S. cerevisiae* and two other yeast species, *Lachancea kluyveri* and *Lachancea thermotolerans*. *S. cerevisiae* genetic diversity is higher in mitochondrial genome compared to nuclear genome in contrast to the two *Lachancea* species. **Figure S3** Inter-clade distances for domesticated and wild lineages. Pink dots represent distances between isolates belonging to domesticated clades, green dots represent distances between isolates belonging to wild clades. Mitochondrial differences in wild clades, do not scale up with the nuclear distance. Domesticate clades show higher diversity at lower nuclear distances compared to the wild clades. **Figure S4** Scatterplot of intra-clades CDS SNPs percentages. Light grey dots represent distances between isolates belonging to distinct clades while dark grey dots represent distances between isolates belonging to the same clade. Dark grey dots circled in black represent isolates belonging to the Mixed origin clade. The line represents the equivalence between the two distances. Dots below the line represent isolate pairs whose mitochondrial distance is lower than the genomic distance. Mixed Origin clade have higher variation in genomic CDS than mitochondrial CDS. Only isolates with complete CDS data have been used (*N*=353). 5. **Figure S5** Comparison of 8-genes networks for mitochondrial and nuclear sequences. Highly divergent lineages (AMH –Taiwanese- and BAG -CHNII-) are not early branching neither in the mitochondrial network (a) nor in the mitochondrial neighbour-joining tree (b), since their sequence diversity is not higher than the typical mitochondrial one. Mixed origin clade isolates confirm a much lower level of nuclear similarity (c and d) compared to the mitochondrial one, in which case their sequences are virtually identical. Finally, three isolates belonging to somehow related clades (BLG -Wine/European-, CCL - Mediterranean oak- and AQM -French Dairy-), while remaining related also in the mitochondrial network, are located further apart (a). **Figure S6** Frequency of different *COX1* introns. The heatmap shows the frequency of the *COX1* introns across the *S. cerevisiae* nuclear clades. Black cells indicate presence in >90% the isolates, white cells indicate <10% in the clade. **Figure S7** Frequency of SNPs at the exon/intron boundary for COX1 and COB genes. Intron/exon boundaries of COX1 (black dots) are enriched for high frequency minor alleles compared with COB intron/exon boundaries (grey dots). **Figure S8** Mitochondrial genome size variation. Size of all genetic elements located on the 250 circularized assemblies, grouped by clade (as described in Peter et al. 2018). **Figure S9** Mitochondrial genome size variation is driven by introns and intergenic regions. Correlation between the length of the mt genome and the cumulative size of the (a) CDS, (b) introns (*p*-value 1.33e-47), (c) intergenic regions (p-value 4.05e-50), (d) intergenic regions and introns. **Figure S10** Structural variation in mitochondrial genomes. Dotplots comparison between the reference genome and the mitochondrial assembly of 4 isolates showing different large inversions. **Figure S11** Copy number of mtDNA across clades. The copy number is calculated as ratio with nuclear genome, to subtract variation derived by the ploidy. Mitochondrial genome copy number is relatively uniform (median ~ 18 copies) with variations in few clades e.g. increased copy number in French Guiana (10.F) isolates.

**Additional file 2 **: **Table S1** Overview of the *S. cerevisiae* collection. Profile id column indicates the unique combination of CDS alleles. Columns reporting the CDS alleles indicate the id of the sequence as reported in the multi fasta database of the unique sequences (http://1002genomes.u-strasbg.fr/files/MitochondrialGenomes/allNonRedundantAlleles.tar.gz). Introns and endonucleases presence/absence is coded 1, for presence, or 0, for absence. NA indicate unavailable sequence. Endonucleases and omega intron were identified by blast search using a cut-off of 90% of identity on 90% of sequence overlap with a reference sequence (see materials and methods). nt in the reference are indicated with the upstream nucleotide position. Positions of the GC cluster refers to the starting position in the reference sequence. Cluster which are absent in the reference sequence are indicated with the first upstream position.

## Data Availability

The datasets supporting the conclusions of this article are included within the article, its additional files and in the 1002 Yeast Genome website dedicated folder, http://1002genomes.u-strasbg.fr/files/MitochondrialGenomes.
