# Supplementary material for: Discordant evolution of mitochondrial and nuclear yeast genomes at population level
Source: BMC Biol. 2020 May 11;18:49. doi: 10.1186/s12915-020-00786-4 (PMC7216626; doi:10.1186/s12915-020-00786-4)
Supplement: Supplementary file 1 — Additional file 1 : Figure S1 Dataset overview. Dataset overview for each clade (named as in Peter et al. 2018) for number of isolates with genome sequenced, complete CDSs assembled and non-redundant profiles. Figure S2 Genetic diversity of nuclear and mitochondrial genes for three yeast species. Distribution of the π values of mitochondrial and nuclear protein coding genes for S. cerevisiae and two other yeast species, Lachancea kluyveri and Lachancea thermotolerans. S. cerevisiae genetic diversity is higher in mitochondrial genome compared to nuclear genome in contrast to the two Lachancea species. Figure S3 Inter-clade distances for domesticated and wild lineages. Pink dots represent distances between isolates belonging to domesticated clades, green dots represent distances between isolates belonging to wild clades. Mitochondrial differences in wild clades, do not scale up with the nuclear distance. Domesticate clades show higher diversity at lower nuclear distances compared to the wild clades. Figure S4 Scatterplot of intra-clades CDS SNPs percentages. Light grey dots represent distances between isolates belonging to distinct clades while dark grey dots represent distances between isolates belonging to the same clade. Dark grey dots circled in black represent isolates belonging to the Mixed origin clade. The line represents the equivalence between the two distances. Dots below the line represent isolate pairs whose mitochondrial distance is lower than the genomic distance. Mixed Origin clade have higher variation in genomic CDS than mitochondrial CDS. Only isolates with complete CDS data have been used (N=353). 5. Figure S5 Comparison of 8-genes networks for mitochondrial and nuclear sequences. Highly divergent lineages (AMH –Taiwanese- and BAG -CHNII-) are not early branching neither in the mitochondrial network (a) nor in the mitochondrial neighbour-joining tree (b), since their sequence diversity is not higher than the typical mitochondrial one. Mixed origin [file 12915_2020_786_MOESM1_ESM.pdf]

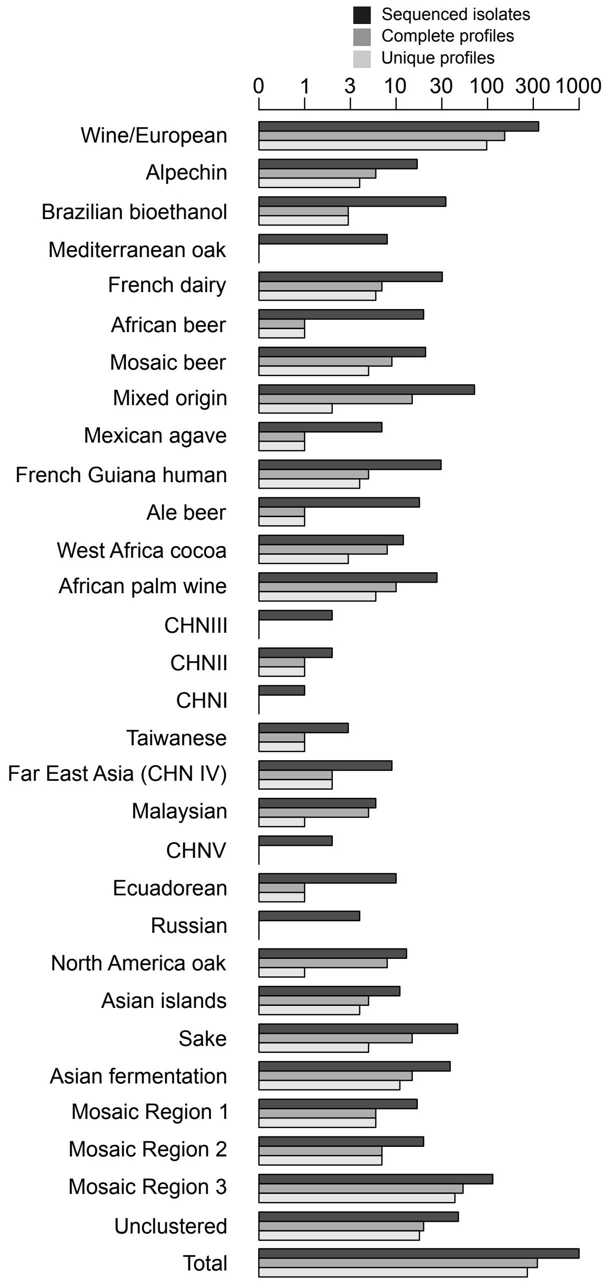

Fig. S1 Dataset overview

Dataset overview for each clade (named as in Peter et al. 2018) for number of isolates with genome sequenced, complete CDSs assembled and non-redundant profiles.

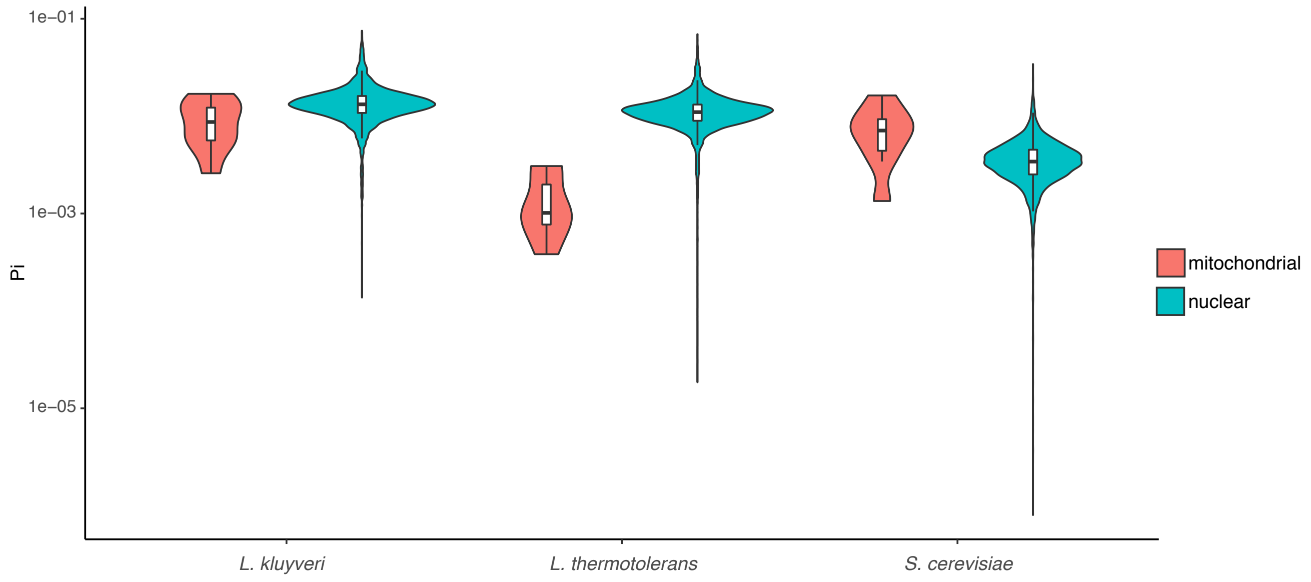

Fig. S2 Genetic diversity of nuclear and mitochondrial genes for three yeast species
Distribution of the π values of mitochondrial and nuclear protein coding genes for *S. cerevisiae* and two other yeast species, *Lachancea kluyveri* and *Lachancea thermotolerans*. *S. cerevisiae* genetic diversity is higher in mitochondrial genome compared to nuclear genome in contrast to the two *Lachancea* species.

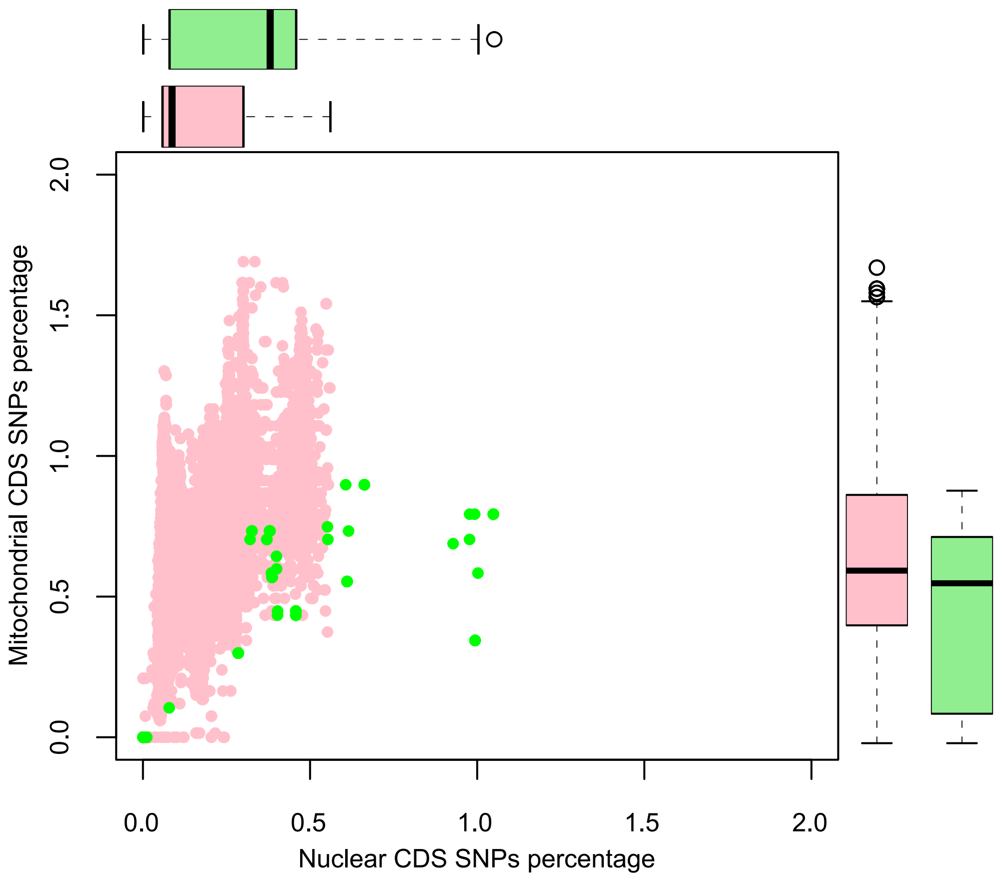

Fig. S3 Inter-clade distances for domesticated and wild lineages

Pink dots represent distances between isolates belonging to domesticated clades, green dots represent distances between isolates belonging to wild clades. Mitochondrial differences in wild clades, do not scale up with the nuclear distance. Domesticate clades show higher diversity at lower nuclear distances compared to the wild clades.

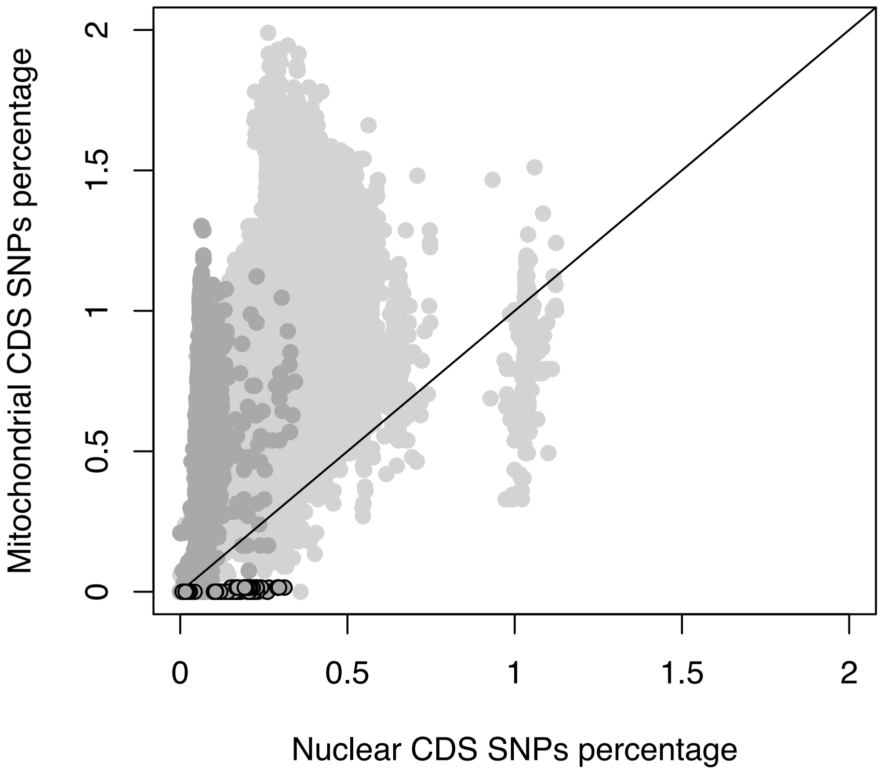

Fig. S4 Scatterplot of intra-clades CDS SNPs percentages

Light grey dots represent distances between isolates belonging to distinct clades while dark grey dots represent distances between isolates belonging to the same clade. Dark grey dots circled in black represent isolates belonging to the Mixed origin clade. The line represents the equivalence between the two distances. Dots below the line represent isolate pairs whose mitochondrial distance is lower than the genomic distance. Mixed Origin clade have higher variation in genomic CDS than mitochondrial CDS. Only isolates with complete CDS data have been used (N=353).

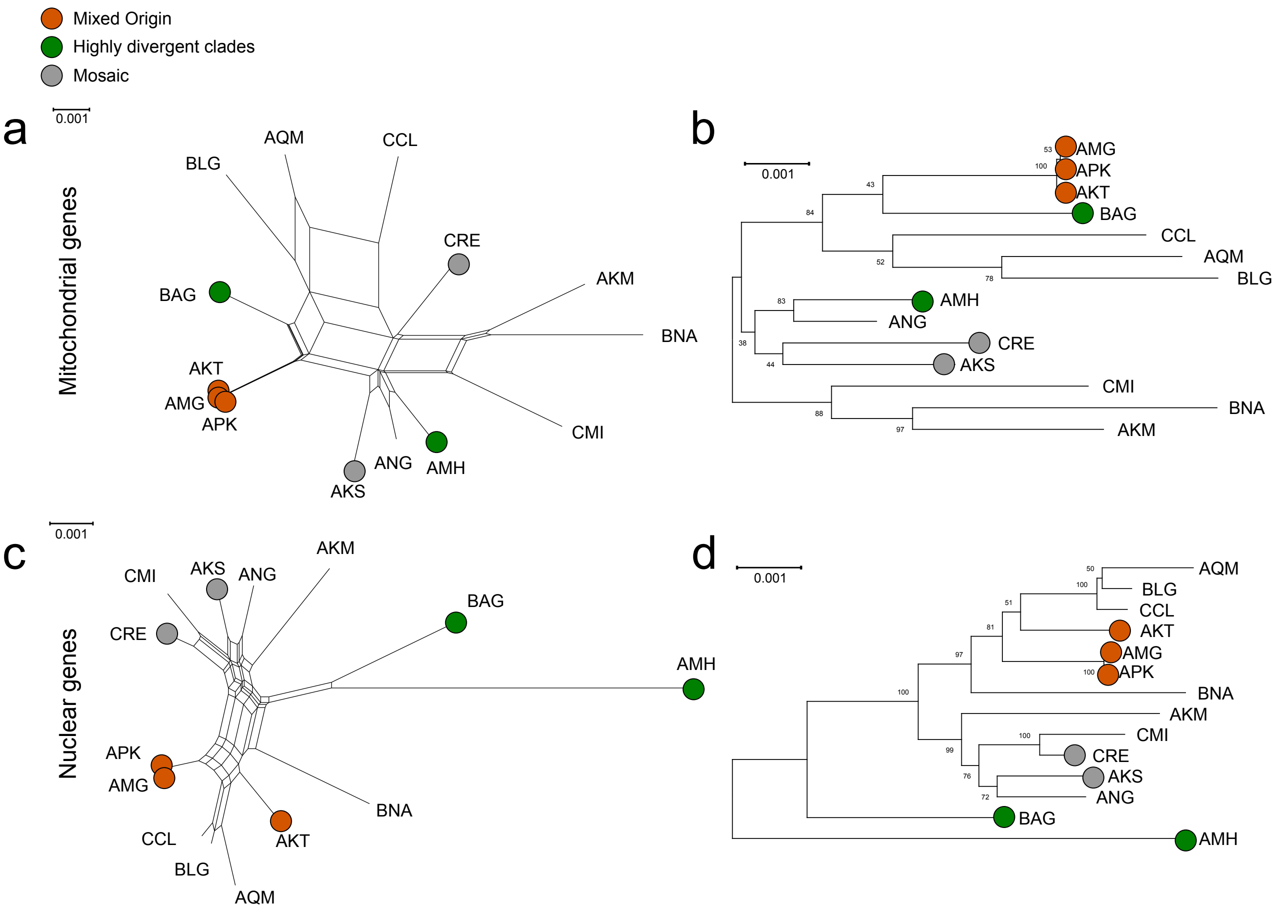

Fig. S5 Comparison of 8-genes networks for mitochondrial and nuclear sequences.
Highly divergent lineages (AMH –Taiwanese- and BAG -CHNII-) are not early branching neither in the mitochondrial network (a) nor in the mitochondrial neighbour-joining tree (b), since their sequence diversity is not higher than the typical mitochondrial one. Mixed origin clade isolates confirm a much lower level of nuclear similarity (c and d) compared to the mitochondrial one, in which case their sequences are virtually identical. Finally, three isolates belonging to somehow related clades (BLG -Wine/European-, CCL -Mediterranean oak- and AQM -French Dairy-), while remaining related also in the mitochondrial network, are located further apart (a).

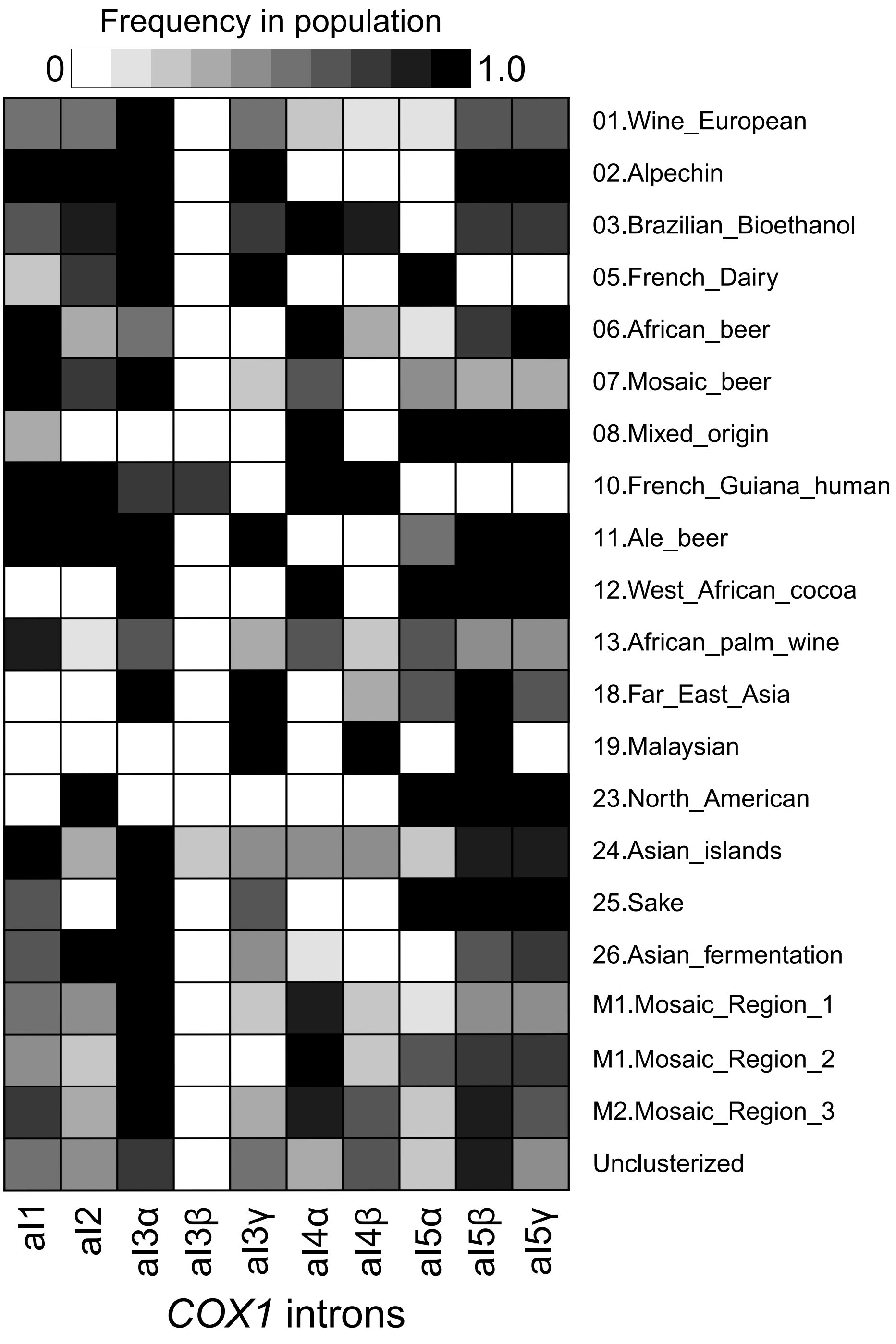

Fig. S6 Frequency of different *COX1* introns

The heatmap shows the frequency of the *COX1* introns across the *S. cerevisiae* nuclear clades. Black cells indicate presence in >90% the isolates, white cells indicate <10% in the clade.

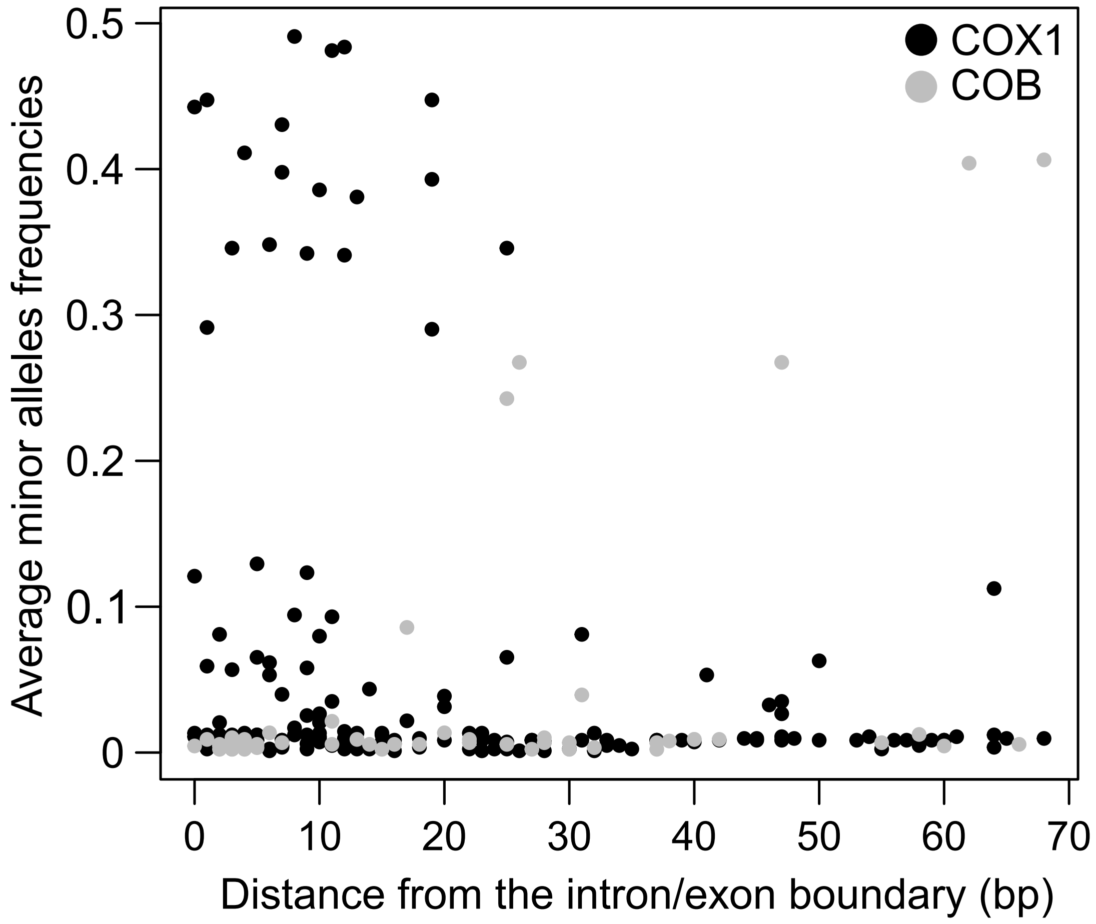

Fig. S7 Frequency of SNPs at the exon/intron boundary for COX1 and COB genes
Intron/exon boundaries of COX1 (black dots) are enriched for high frequency minor alleles compared with COB intron/exon boundaries (grey dots).

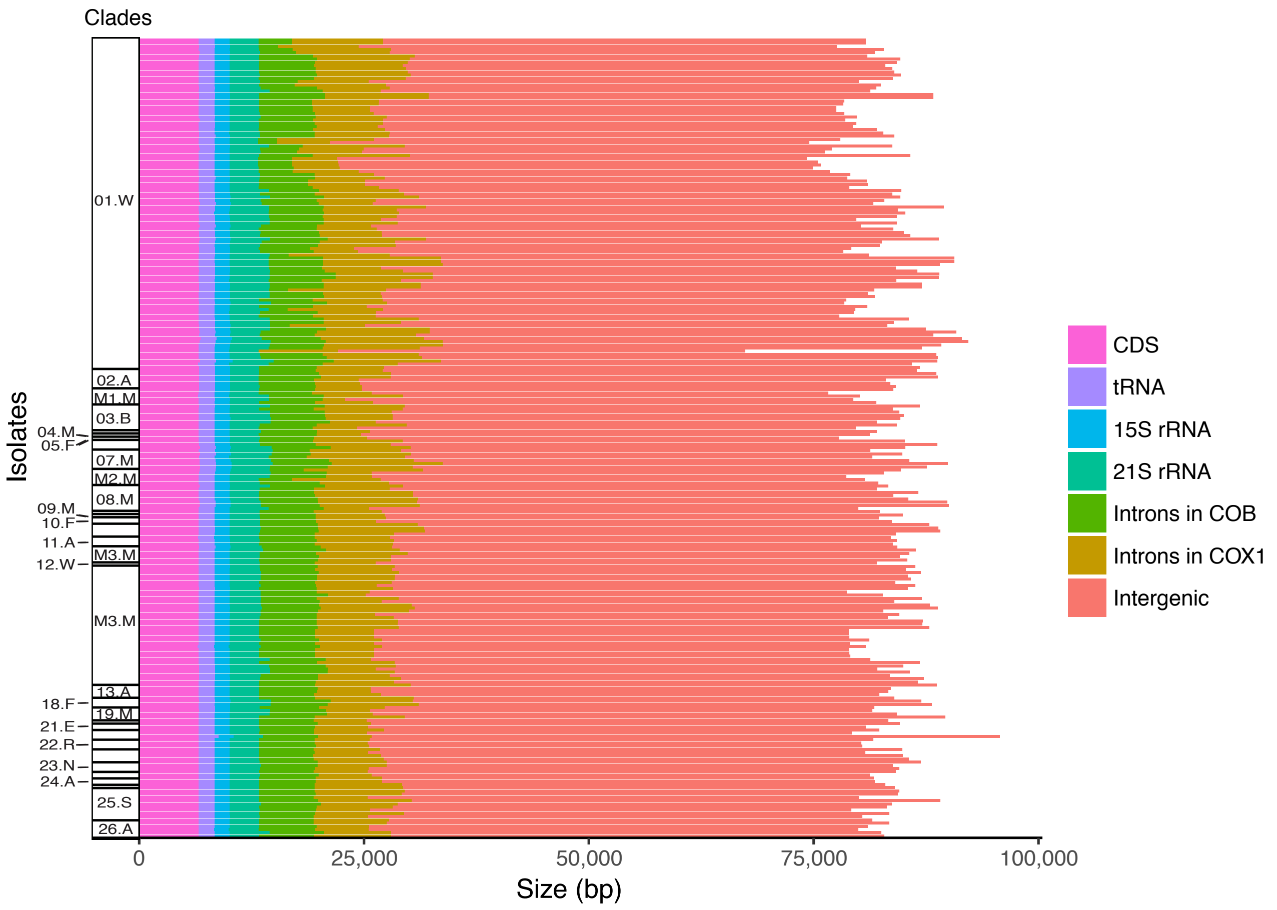

Fig. S8 Mitochondrial genome size variation

Size of all genetic elements located on the 250 circularized assemblies, grouped by clade (as described in Peter et al. 2018).

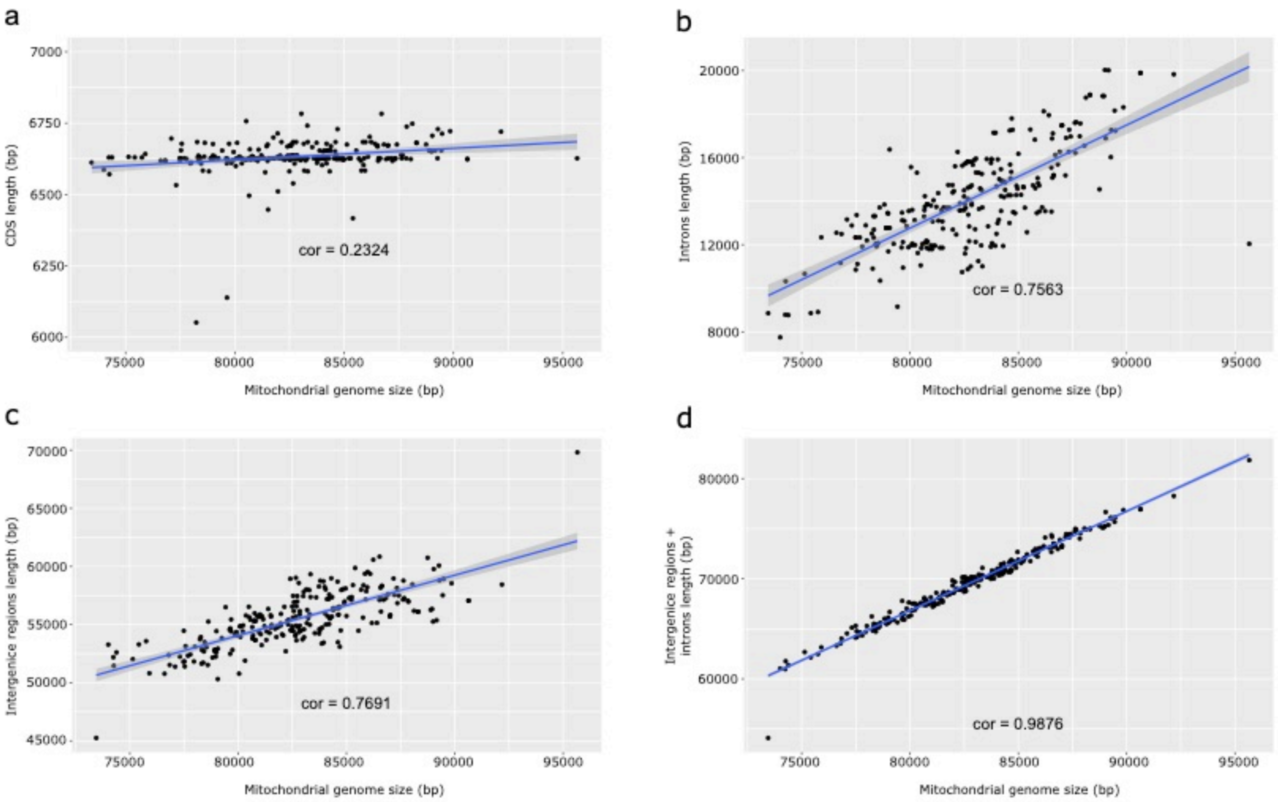

Fig. S9 Mitochondrial genome size variation is driven by introns and intergenic regions
Correlation between the length of the mt genome and the cumulative size of the (a) CDS, (b) introns (p-value 1.33e-47) , (c) intergenic regions (p-value 4.05e-50), (d) intergenic regions and introns.

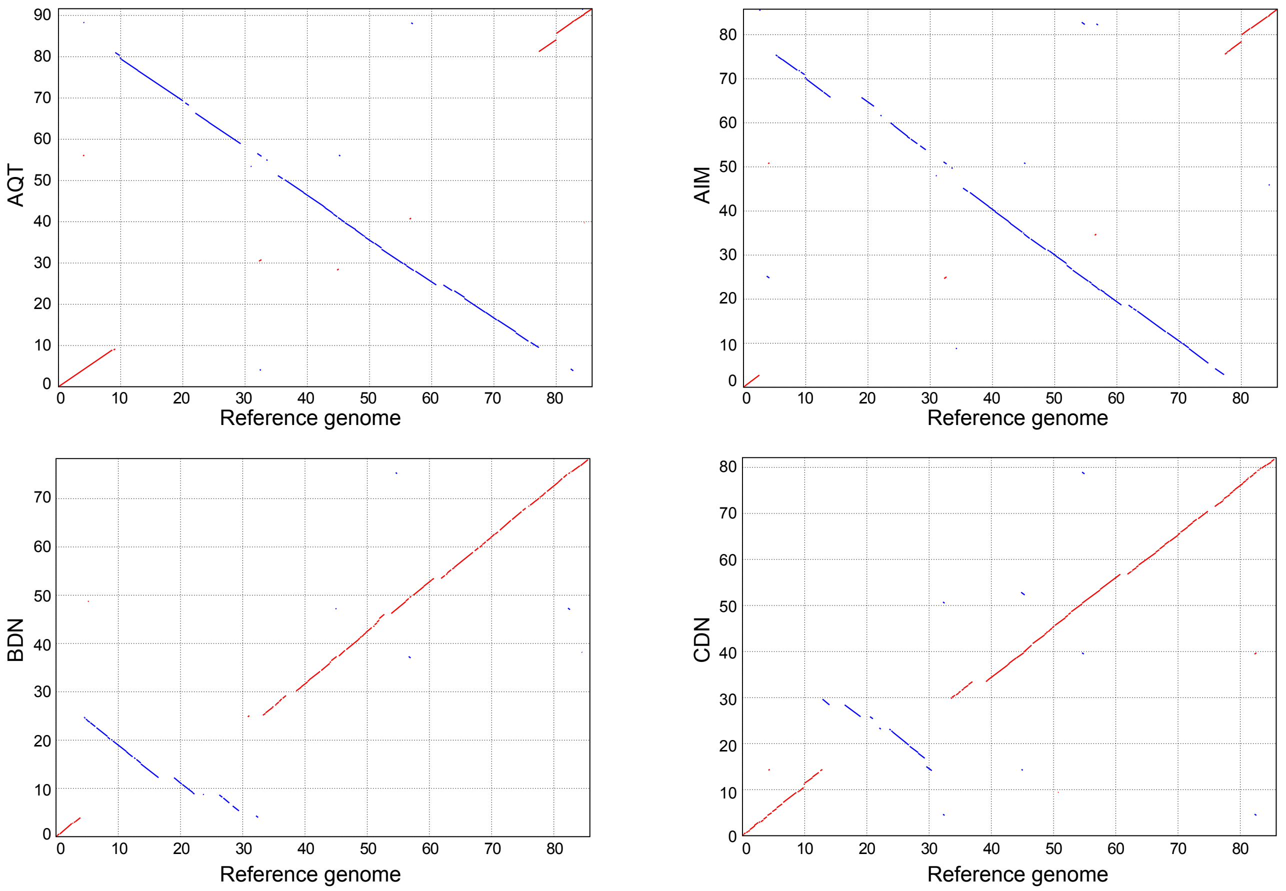

Fig. S10 Structural variation in mitochondrial genomes

Dotplots comparison between the reference genome and the mitochondrial assembly of 4 isolates showing different large inversions.

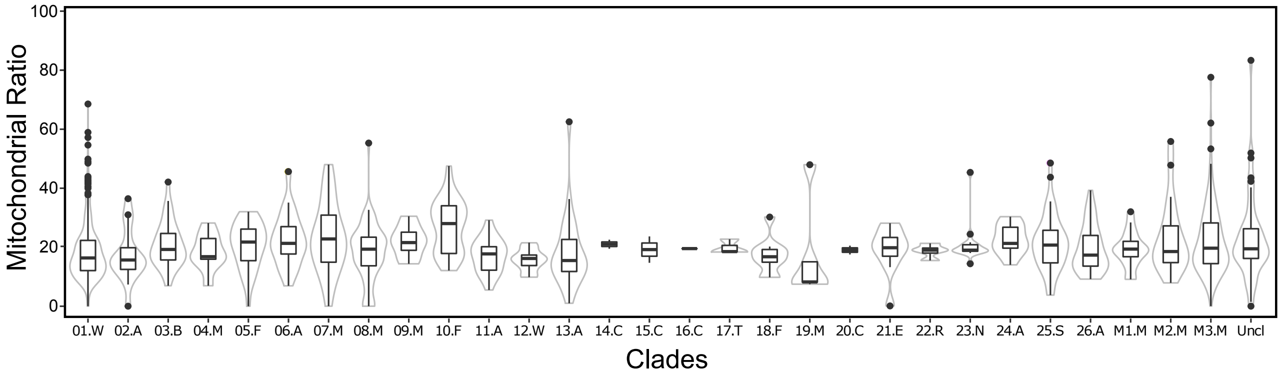

Fig. S11 Copy number of mtDNA across clades

The copy number is calculated as ratio with nuclear genome, to subtract variation derived by the ploidy. Mitochondrial genome copy number is relatively uniform (median ~ 18 copies) with variations in few clades *e.g.* increased copy number in French Guiana (10.F) isolates.
